# Radiation recall reaction causing cardiotoxicity

**DOI:** 10.1186/1532-429X-16-25

**Published:** 2014-04-22

**Authors:** Sofia Carolina Masri, Andrew James Misselt, Arkadiusz Dudek, Suma H Konety

**Affiliations:** 1 Department of Medicine, Division of Cardiology, University of Washington, 1959 NE Pacific Street, BOX 356422, Seattle, WA, USA; 2 Department of Medicine, Division of Cardiology, University of Minnesota, Minneapolis, MN, USA; 3 Department of Diagnostic Radiology, University of Minnesota, Minneapolis, MN, USA; 4 Department of Medicine, Division of Hematology, Oncology, Transplantation, University of Minnesota, Minneapolis, MN, USA

## Abstract

Radiation recall phenomenon is a tissue reaction that develops within a previously irradiated area, precipitated by the subsequent administration of certain chemotherapeutic agents. It commonly affects the skin, but can also involve internal organs with functional consequences. To our best knowledge, this phenomenon has never been reported as a complication on the heart and should be consider as a potential cause of cardiotoxicity.

## Background

Radiation recall reaction (RRR) refers to an inflammatory reaction within a previously treated radiation field. The reaction occurs in response to the combined effect of radiotherapy and a second precipitating agent, mainly with chemotherapeutic drugs. We would like to describe a case of RRR that involved the heart.

## Case presentation

We present a 56-year old man with a prior history of adenocarcinoma of the lung with metastasis to cervical lymph nodes and brain diagnosed in 2006. He was initially treated with chemotherapy in 2006. Subsequently, he required stereotactic radiation to the chest and cervical lymph nodes with a total dose of 7000 CGy. Since his initial therapies, he has received multiple chemotherapy treatments including cisplatin, docetaxel, bevacizumab, erlotinib, and gemcitabine. Most recently, he was started on sorafenib.

Five month later, he presented with chest pain. No significant electrocardiographic changes were present and his troponin level was normal. An echocardiogram showed mildly decreased left ventricular systolic function with an ejection fraction of 45%. The anterior wall was hypokinetic and there was a small pericardial effusion.

**Figure 1:**
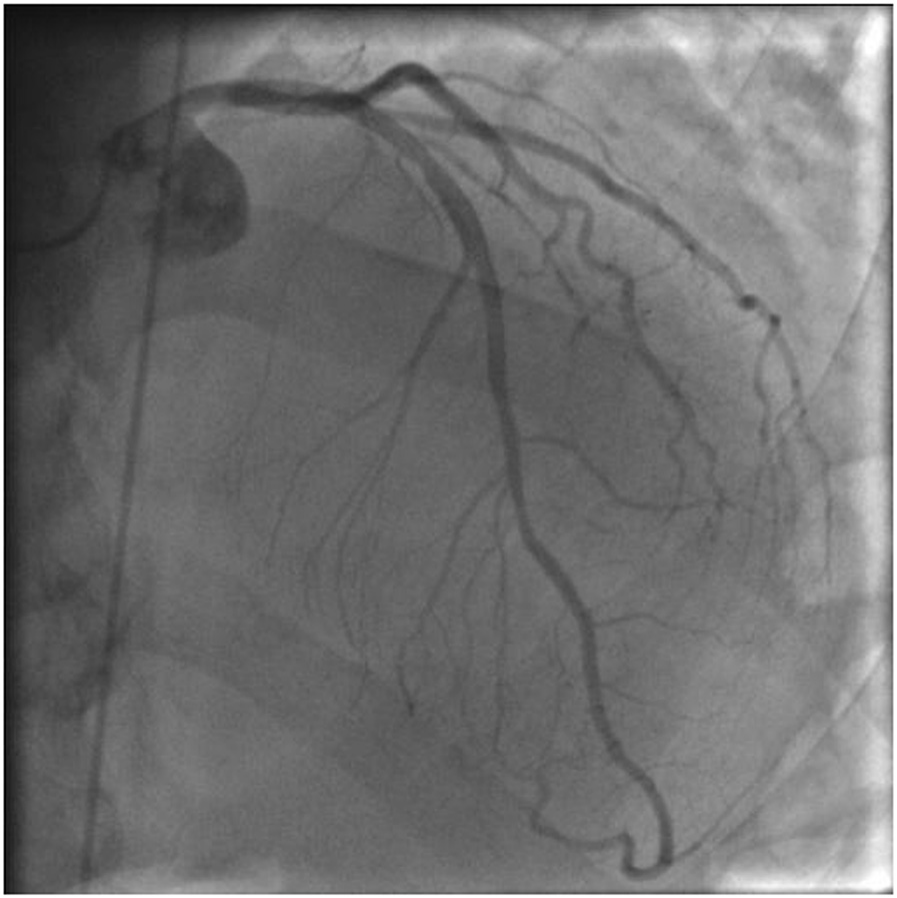
Left coronary angiogram, showing the left anterior descending artery (LAD) and left circumflex artery (LCX).

A coronary angiogram was performed which demonstrated no significant coronary artery disease (Figure 1).

**Figure 2:**
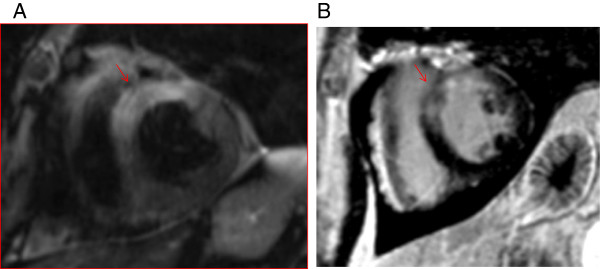
**CMR short axis images of the mid left ventricle.** T2-weighted image **(A)** demonstrates bright myocardium (arrow) suggesting edema in the anterior and anteroseptal wall. Late gadolinium enhancement **(B)** (arrow) corresponds with the territories of bright myocardium on T2-weighted imaging, suggesting edema or a combination of edema and fibrosis.

Cardiovascular magnetic resonance (CMR) was then performed, which demonstrated increased signal onT2 weighted images in the basal and mid anterior wall, indicative of edema (Figure 2A). Using late gadolinium enhancement (LGE) imaging, enhancement was identified in the same portions of the basal and mid anterior wall (Figure 2B).

**Figure 3:**
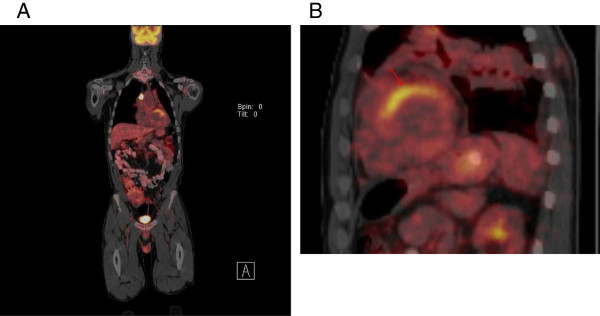
**Whole-body FDG PET image in fasting condition shows FDG accumulation in the submandibular region, mediastinal, and right hilum lymph nodes and the heart involving basal and mid anterior wall (A).** Short-axis image of fasting FDG-PET of the heart with FDG accumulation in the anterior and anteroseptal wall **(B)**.

**Figure 4:**
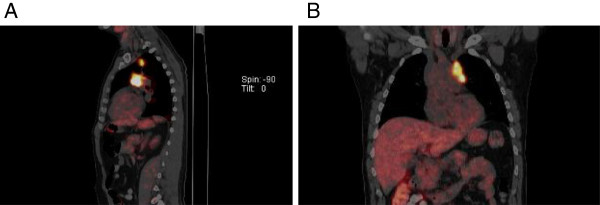
**Whole-body FDG PET image in a fasting condition shows FDG accumulation in multiple prevascular, left hilar and subcarinal lymph nodes.** There is no myocardial uptake of FDG. Sagital image of fasting FDG-PET **(A)**. Coronal image of fasting FDG-PET **(B)**.

**Figure 5:**
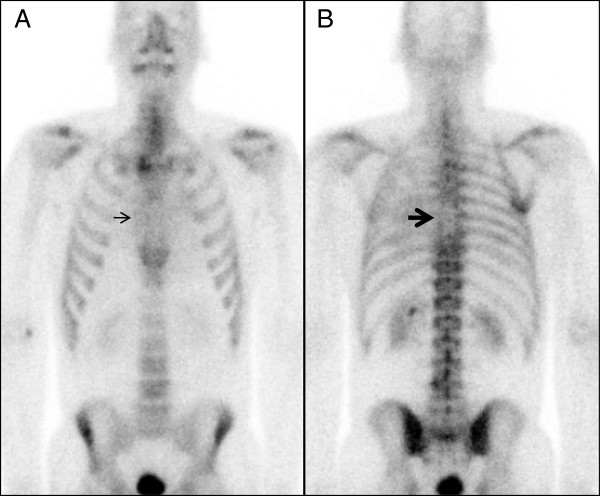
Whole body nuclear bone scan revealed a photopenic area in the mid thorax.

A FDG-PET /CT performed 3 months prior to the patient’s clinical presentation of chest pain (about 2 months into sorafenib therapy), revealed regional uptake of FDG in the same the basal and mid anterior wall segments (Figure 3A and B). These findings were new compared to a FDG-PET/CT scan performed 2 years earlier (about 1 year after the initial radiation therapy) which had showed no myocardial uptake of FDG (Figure 4A, B). Whole body nuclear bone scan revealed a photopenic area in the mid thorax (Figure 5), attributable to prior radiation therapy.

Interestingly, the abnormal myocardial wall segments demonstrated on FDG-PET/CT, echocardiography and CMR also corresponded to the previously irradiated area. Due to these findings, sorefanib was discontinued. He had no recurrent chest pain.

## Discussion

Radiation recall reaction (RRR) refers to an inflammatory reaction within a previously treated radiation field. The reaction occurs in response to the combined effect of radiotherapy and a second precipitating agent. The second precipitating agent has been observed mainly with chemotherapeutic drugs. It can occur months or even many years after irradiation, suggesting that the mechanism may be different from radiosensitization [1]. Skin is the major site of radiation recall toxicity. But, the phenomenon has also been described in other organs including the lung, digestive tract, muscle, and central nervous system. However, to the best of our knowledge radiation recall toxicity has never been reported in the heart.

We believe our case of a 56 year-old man with new onset chest pain in the setting of remote radiation therapy and ongoing chemotherapy likely represents an instance of radiation recall myocarditis. Features such as acute onset reduced ejection fraction with regional wall motion abnormalities, regional inflammation and edema on FDG-PET/CT and T2 weighted CMR, and regional enhancement on LGE-CMR may result from a variety of causes, including ischemic heart disease. Our patient underwent coronary angiography and had no evidence of obstructive coronary disease. Other possibilities might include infiltrative disease like cardiac sarcoidosis or myocarditis [2]. However, it would be highly improbable for our patient to develop de novo infectious myocarditis or sarcoid in precisely the same anatomic distribution as the previous radiation field.

We propose that the diagnosis of radiation recall reaction myocarditis induced by chemotherapy is established in our case by a history of chemotherapy after thoracic radiotherapy, with CMR and FDG-PET/CT showing evidence of inflammation in the irradiated areas of the myocardium. When radiation therapy is followed by chemotherapy, subclinical damage from irradiation can be unmasked or potentiated and ultimately manifest clinically as RRR.

Taxanes and anthracyclines have been reported to be responsible for 20% and nearly 30% of radiation recall reaction, respectively [3]. The inciting agents observed in radiation recall phenomenon previously reported included taxanes, anthracyclines, gemcitabine, etoposide, vinorelbine and erlotinib [3-5]. Sorafenib is a small molecule tyrosine kinase inhibitor of vascular endothelial growth factor (VEGF) and platelet-derived growth factor (PDGF) receptors, increasingly used in advanced solid organ treatment and it has recently been implied in this phenomenon [6]. The range of chemotherapeutic drugs associated with the phenomenon is diverse and no common features or characteristics of the drugs have been identified. It is not clear whether RRR are a class effect or related to the drug dose or regimen. Reactions have occurred with a range of dosages of different drugs. The mechanism of this phenomenon is not clear, though it may be related to increased local vascular permeability caused by radiotherapy. In our case, this phenomenon was likely exacerbated by subsequent administration of sorenafib and resultant endothelial dysfunction from its inhibitory effects on VEGF receptor. The mechanism is unknown but may involve impaired of the angiogenesis leading to a decrease in the microvessels (a process known as capillary rarefaction) or endothelial dysfunction associated with a decrease in nitric oxide [7]. The reported time interval between radiation and subsequent chemotherapy leading to RRR is variable from days to years. Current treatment strategies include the withdrawal of the causative agents and administration of systemic steroids [8].

## Conclusion

Radiation recall phenomenon is a tissue reaction that develops within a previously irradiated area, precipitated by the subsequent administration of certain chemotherapeutic agents. It commonly affects the skin, but can also involve internal organs with functional consequences. While undoubtedly rare and previously unreported, radiation recall phenomenon should be considered as a potential cause of cardiotoxicity in the appropriate context.

## Consent

This case report was approved by the IRB of University of Minnesota Medical Center and the requirement for the individual consent was waived based on retrospective design.

## Competing interest

Drs. Masri, Misselt, Dudek, Konety report no competing interest.

## Authors’ contributions

SCM: conception and design, acquisition, analysis and interpretation of data, drafting of manuscript; AJM: critical and intellectual revision of manuscript; AD: critical and intellectual revision of manuscript; SHK: critical and intellectual revision of manuscript. All authors read and approved the final manuscript.
